# Cutaneous vasculitis in systemic lupus erythematosus: epidemiology and risk factors over a 20-year follow-up

**DOI:** 10.1093/rheumatology/keae672

**Published:** 2024-12-11

**Authors:** Ahmed Saleh, Chee-Seng Yee, Aba Acquah, Caroline Gordon, John A Reynolds

**Affiliations:** Department of Inflammation and Ageing, School of Infection, Inflammation and Immunology, College of Medicine and Health, University of Birmingham, Birmingham, UK; Rheumatology Department, Faculty of Medicine, Mansoura University, Mansoura, Arab Republic of Egypt; Doncaster and Bassetlaw Teaching Hospitals NHS Foundation Trust, Doncaster, UK; Department of Inflammation and Ageing, School of Infection, Inflammation and Immunology, College of Medicine and Health, University of Birmingham, Birmingham, UK; Department of Inflammation and Ageing, School of Infection, Inflammation and Immunology, College of Medicine and Health, University of Birmingham, Birmingham, UK; Rheumatology Department, Sandwell and West Birmingham NHS Trust, Birmingham, UK; Department of Inflammation and Ageing, School of Infection, Inflammation and Immunology, College of Medicine and Health, University of Birmingham, Birmingham, UK; Rheumatology Department, Sandwell and West Birmingham NHS Trust, Birmingham, UK

**Keywords:** cutaneous vasculitis, SLE, lupus, classic BILAG, SLICC/ACR damage index

## Abstract

**Objectives:**

Cutaneous vasculitis (CV) is common in SLE, but the epidemiology and risk factors remain unclear. We aimed to identify the trends and risk factors for CV in patients with SLE over a period of 20 years.

**Methods:**

The Birmingham Lupus Cohort is an observational longitudinal cohort of SLE patients. Patients were enrolled within 3 years of meeting their fourth ACR criterion. Disease activity, laboratory test results and treatment records were collected. A multivariable shared frailty Cox proportional hazard model was used to identify clinical, laboratory and treatment-related variables associated with the development of CV.

**Results:**

We included 392 patients: 95.7% were female. The median (interquartile range) duration of follow-up was 9.2 (5.1–14.7) years. CV occurred in 27% of SLE patients, of whom 43.3% had two or more CV events. This study demonstrated a marked decline in the incidence rates of CV, decreasing from 34.4% (95% CI 29.7, 39.3) during the first 3 years after enrolment to 2.1% (95% CI 0.05, 11.5) after 18 years of follow-up. Development of CV was associated with RP, constitutional, mucocutaneous, musculoskeletal, haematological and cardiovascular involvement, anti-Sm antibodies, anti-dsDNA, and hypocomplementemia. However, the use of AZA and antimalarials was inversely associated with the development of CV. Patients with CV were more likely to develop at least one item of organ damage.

**Conclusions:**

The incidence rates of CV in SLE decreased over the follow-up period and CV is associated with defined clinical, serological and treatment-related factors.

Rheumatology key messages

- The majority of cutaneous vasculitis (CV) episodes in patients with SLE occurred early in the disease course.
- Over 40% of patients with CV had more than one episode.
- The development of CV is associated with increased overall lupus disease activity and organ damage.

## Introduction

SLE is an uncommon systemic autoimmune disease which predominantly affects females. This disorder is characterized by the production of autoantibodies and immune complexes deposition in multiple organs [1]. While the exact prevalence of SLE varies across different populations, it is estimated to affect ∼15–110 cases per 100 000 persons worldwide [2]. It is more prevalent and severe in people from Hispanic, African and Asian backgrounds than a White background [3]. SLE displays a wide range of clinical manifestations, from mild cutaneous involvement to severe life-threatening multiorgan failure [4].

Blood vessel involvement can occur in SLE and is associated with significant morbidity and mortality [5]. Lupus vasculitis typically affects small blood vessels, and whilst medium-sized vessels can also be affected, large blood vessels are rarely impacted [6]. Previous studies suggested that SLE patients with vasculitis are typically male, and have earlier disease onset and longer disease duration [7]. Vasculitis can manifest in various clinical presentations, dependent on the size and location of vessels involved (typically the skin or internal organs). The severity of lupus vasculitis can vary, ranging from mild to life-threatening [3].

Cutaneous vasculitis (CV) is the most common form of vasculitis observed in patients with SLE. Typical features of cutaneous small vessel vasculitis include ulcers, punctate lesions, palpable purpura and erythema with necrosis [8]. According to the classic BILAG index, CV is classified into (i) major CV, which includes ulceration with infarction, and (ii) minor CV, which includes nailfold vasculitis, digital vasculitis, purpura and urticaria [9].

Only a few studies have reported the epidemiology of, and risk factors for, CV in patients with SLE. Kallas *et al.* studied a large multi-ethnic American cohort from 1987 to 2019, involving 2580 SLE patients, of whom 449 (17.4%) patients had CV [10]. However, a Spanish study between 1980 and 2004 by Ramos-Casals *et al.* reported that 68 (10.1%) of 670 lupus patients had developed CV [8]. This included erythematous punctate lesions on the fingertips and palms in 36%, purpura in 25%, ischaemic lesions in 14%, erythematous papules/macules in 14%, urticarial lesions in 11% and nodular lesions in 5%. Several studies have documented that CV was positively associated with anti-dsDNA, hypocomplementemia [7, 10], anti-Ro/SSA, anti-La/SSB and disease activity [8, 11].

Although CV represents a significant systemic manifestation in SLE, there are limited data regarding the incidence, prevalence and underlying risk factors. The aim of this study was to identify the changes in epidemiology and the potential risk factors for CV in SLE patients over a period of 20 years, in a multi-ethnic, UK-based inception cohort.

## Patients and methods

The Birmingham SLE Cohort is a longitudinal observational cohort of ∼600 patients with SLE, established in 1989, that have been routinely followed at Birmingham City Hospital (now part of Sandwell and West Birmingham NHS Trust) including some patients seen at Queen Elizabeth Hospital (University Hospital Birmingham NHS Foundation Trust). This study received ethical approval from Wales REC 6 (Ref. 20/WA/0228) and City Hospital ethics committee (LREC 01/04/243) and the South Birmingham ethics committee (LREC 5749), in accordance with the guidelines of the Declaration of Helsinki. All patients enlisted in the study provided a signed, informed consent form. All patients fulfilled the 1997 ACR Updated Classification Criteria for SLE [12]. Inception patients were defined as those recruited within 3 years of achieving their fourth ACR criterion.

At each medical consultation, disease activity was assessed by the classic BILAG index which includes a specific vasculitis domain and any changes in medication were recorded using the British Lupus Integrated Prospective System (BLIPS, from ADS-Limathon) [9, 13]. Damage was evaluated using the SLICC/ACR damage index (SDI) at least once a year [14]. In addition, demographic background, ACR classification criteria, serological test results and smoking were recorded. Data reported here were collected between 1989 and 2013. Damage and death data were censored at 10-year follow-up.

### Data collection

Clinical data was recorded using the classic BILAG index [13]. CV was defined using the vasculitis system in the classic BILAG index (which is separate from the mucocutaneous system). It classifies CV into (i) major CV, which includes ulcerations with infarction, and (ii) minor CV, which includes nailfold vasculitis, digital vasculitis, purpura and urticaria [9].

Routinely collected laboratory data, including ANA, anti-dsDNA, anti-Sm, anti-Ro/SSA, anti-La/SSB, anti-RNP, C3 and C4, were obtained through standardized testing methods in accordance with established clinical protocols at each hospital. Additionally, immunosuppressive medications were documented as described previously [15].

### Statistical analysis

Descriptive analysis was used to summarize the baseline characteristics. Categorical variables were expressed as percentage, and continuous variables as median with interquartile range. Categorical variables were compared using the χ^2^ test. A CV event was defined as an episode if it scored ‘new’, ‘improved’, ‘same’ or ‘worse’ on the BILAG index provided that the manifestation was recorded as ‘not present’ at the previous visit. When calculating incidence rates, each episode was counted as a separate occurrence under the above rule. The cumulative incidence for the first CV event per patient was estimated using two methods. First, we estimated the cumulative incidence function (CIF) as 1—survivor function (Kaplan–Meier) at the 3-year time points. Second, we calculated the CIF using a competing risk model with the stcrreg function in Stata software. The competing risks were the occurrence of a CV event and death [16]. Incidence rates and cumulative incidence of CV were calculated in 3-year periods. This periodic assessment aimed to capture changes in incidence and minimize the potential impact of prolonged follow-up period for inception patients enrolled in the study.

A multivariable Cox Proportional Hazard shared frailty model was developed to identify clinical, laboratory and treatment variables associated with the development of CV. Unlike a standard Cox model, the shared frailty correction accounts for the dependence between recurrent events and the effects of unobserved heterogeneity on outcomes [17]. By analysing each CV episode as a separate occurrence and incorporating individual level heterogeneity, the model provides hazard estimates which account for the correlated nature of recurrent events within individuals, through the inclusion of a random effect [18]. The hazard ratio (HR) can be interpreted as the relative risk of experiencing a CV event accounting for the effect of covariates on the hazard, while adjusting for the shared frailty (random effect) [19].

Covariates included in the multivariable model were prespecified based on our knowledge base and clinical experience. For each patient, the time between CV episodes was included in the model as the time variable. The proportional hazard assumptions were assessed by Schoenfeld residuals test and variables that violated proportional hazards were included as time-varying covariates (TVC) in a sensitivity analysis [20]. Medications used at the time of fewer than 10 CV events were excluded from the multivariable model. Data were analysed with Stata for windows version 17 (Stata Corporation, College Station, TX, USA).

## Results

We included a total of 392 inception SLE patients of whom 375 (95.7%) were female. There were 213 (54.3%) White, 72 (18.4%) African or Caribbean, 82 (20.9%) South Asian, 9 (2.3%) East Asian patients and 11 (2.8%) from other ethnic backgrounds. The median [interquartile range (IQR)] duration of follow-up was 9.2 (5.1–14.7) years and the median age at enrolment was 33 (26–44) years (Table 1). The medications used at the time of CV events are summarized in Table 1.

**Table 1.** Demographic characteristics

Patient characteristic	Patients *n* = 392
Female sex, *n* (%)	375 (95.7)
Ethnic group, *n* (%)	
African or Caribbean	72 (18.4)
South Asian	82 (20.9)
White	213 (54.3)
East Asian	9 (2.3)
Others	11 (2.8)
Age at recruitment, median (IQR), years	33.0 (26, 44)
Duration of follow-up, median (IQR), years	9.2 (5.1, 14.7)
Clinical characteristics at study entry	
ACR criteria, *n* (%)	
Positive ANA	382 (97.4)
Arthritis	343 (87.5)
Haematological disorder	265 (67.6)
Immunological disorder	237 (60.4)
Oral ulcers	201 (51.2)
Photosensitivity	165 (42.0)
Malar rash	134 (34.1)
Serositis	119 (30.3)
Renal disorder	71 (18.1)
Discoid rash	49 (12.5)
Neurological disorder	28 (7.1)
Classic BILAG index (active disease = grade A or B)	
Musculoskeletal	144 (36.7)
Mucocutaneous	120 (30.6)
Haematological	100 (25.5)
Renal	54 (13.7)
Cardiovascular and respiratory	40 (10.2)
General	38 (9.6)
Vasculitis	30 (7.6)
Neurological	30 (7.6)
Medications in use at the time of CV event, *n* (%)	CV events *n* = 264
Prednisolone	
Low dose (≤7.5 mg/day)	32 (12.1)
Medium dose (>7.5 mg—30 mg/day)	137 (51.8)
High dose (>30 mg/day)	36 (13.6)
Antimalarial	114 (43.3)
MMF	22 (8.3)
CYC	26 (9.8)
MTX	5 (1.8)
AZA	69 (26.1)
Ciclosporin	9 (3.4)
Rituximab	7 (2.6)

CV: cutaneous vasculitis; IQR: interquartile range.

At study entry, apart from ANA positivity, arthritis was the most prevalent ACR criterion, with 343 (87.5%) patients, followed by haematological disorders in 265 (67.6%) patients. In addition, 367 (66.0%) patients had active disease at enrolment, as indicated by an ‘A’ or ‘B’ score in the classic BILAG Index. The most common active disease domains were musculoskeletal in 144 (36.7%), mucocutaneous in 120 (30.6%) and haematological in 100 (25.5%) (Table 1).

CV occurred in 106/392 (27%) patients with SLE over the study period, without accounting for those lost to follow-up. Among these 106 patients, a total of 264 CV events were documented, of which 221 were minor CV and 43 were major CV. Forty-six of 106 (43.3%) patients with CV had two or more CV events, of whom 11 (10.3%) had six or more CV episodes. The maximum number of CV episodes observed in a single patient was 16. The median (IQR) time between episodes was 1386 (497–2430) days. 12/21 (57.1%) of African or Caribbean patients with CV had more than one CV episode, compared with 22/54 (40.7%) of White and 10/25 (40.0%) of South Asians, although this was not statistically significant (*P* = 0.342).

**Figure 1.**
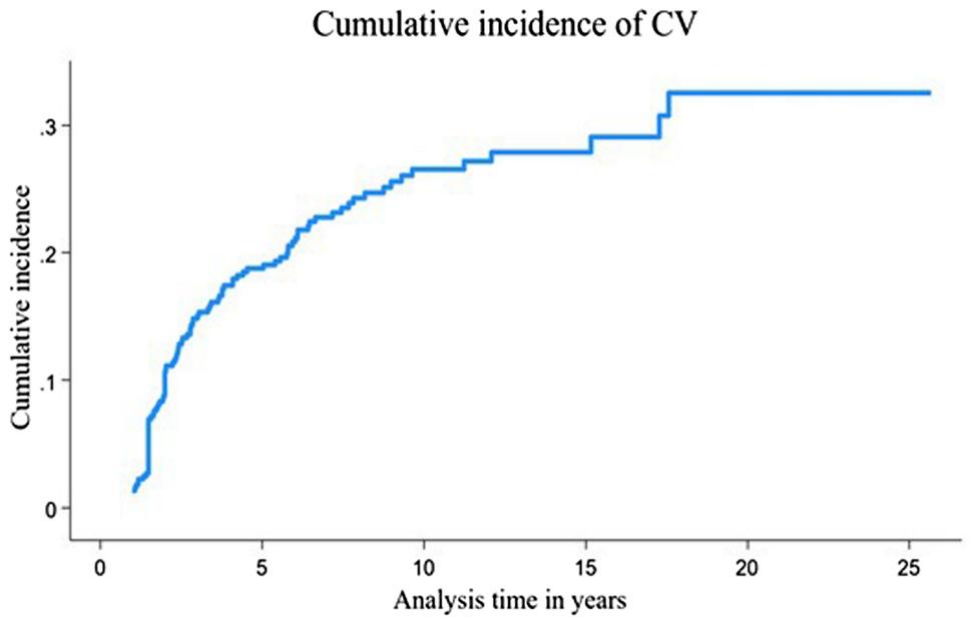
Cumulative incidence of CV over the follow-up period. The cumulative incidence function was calculated using a competing risk model. The competing risks were the occurrence of first CV event per patient and censorship (death). There was a significant increase in the cumulative incidence during the first 9 years of follow-up, followed by a steady rise until the end of the study. CV: cutaneous vasculitis

There was a decrease in the incidence of CV over the follow-up period. Within the first 3 years following enrolment, the incidence rate of CV was 120.0 (95% CI 101.2, 142.2) per 1000 person-years; however, after 18 years of follow-up, the incidence rate decreased to 7.6 (95% CI 0.1, 54.1) per 1000 person-years (Table 2). The cumulative incidence of CV at 3, 9 and 18 years was estimated at 0.17 (95% CI 0.13, 0.21), 0.27 (95% CI 0.23, 0.32) and 0.33 (95% CI 0.27, 0.40), respectively (see Supplementary Table S1, available at *Rheumatology* online). To account for changes in patient numbers, the cumulative incidence of CV with death as a competing risk was calculated (Fig. 1). There was an increase in the cumulative incidence during the first 9 years of follow-up, followed by a steady rise until the end of the study.

**Table 2.** The cumulative incidence of minor and major CV in triannual intervals in the cohort over the period of follow-up

Period of follow-up, years	Minor CV cumulative incidence % (95% CI)	Major CV cumulative incidence % (95% CI)	CV cumulative incidence % (95% CI)
0–3	29.8 (25.3, 34.6)	4.5 (2.7, 7.1)	34.4 (29.7, 39.3)
3–6	15.4 (11.7, 19.7)	2.6 (1.2, 4.9)	18.0 (14.1, 22.5)
6–9	10.3 (7.0, 14.6)	3.3 (1.5, 6.2)	13.7 (9.8, 18.3)
9–12	6.9 (3.8, 11.4)	2.9 (1.1, 6.3)	9.9 (6.1, 14.9)
12–15	3.5 (1.1, 8.1)	0.7 (0.01, 3.9)	4.2 (1.5, 9.0)
15–18	3.1 (0.6, 9.0)		3.1 (0.6, 9.0)
>18	2.1 (0.05, 11.5)		2.1 (0.05, 11.5)

CV: cutaneous vasculitis.

The results of univariate analysis using shared frailty Cox proportional hazards models are summarized in Table 3. In a multivariable analysis, there was a significant association between CV and: RP [HR 3.28 (95% CI 2.34, 4.61)], anti-dsDNA [HR 1.40 (95% CI 1.01, 1.97)], anti-Sm antibodies [HR 2.37 (95% CI 1.31, 4.31)] and hypocomplementemia [HR 1.85 (95% CI 1.31, 2.63)]. All systems of the classic BILAG index (after exclusion of the vasculitis system) were associated with CV except for the neuropsychiatric domain (Table 3). The use of AZA [HR 0.62 (95% CI 0.42, 0.91)] and antimalarials [HR 0.65 (95% CI 0.46, 0.92)] was inversely associated with the development of CV (Table 3). Although the antimalarial medication and BILAG haematological system variables violated the proportional hazards assumption, a sensitivity analysis using these variables as TVC did not significantly improve model fit (Supplementary Tables S2 and S3, and Figs S1 and S2, available at *Rheumatology* online).

**Table 3.** Clinical, laboratory and treatment-related risk factors of development of CV

Variables	Univariate Cox regression HR (95% CI)	Multivariable Cox regression HR (95% CI)
Sex: male	0.35 (0.11, 1.08)	0.57 (0.16, 2.00)
Age	0.92 (0.91, 0.94)	0.95 (0.93, 0.97)
Ethnicity		
White	Ref	
African or Caribbean	2.09 (1.12, 3.89)	0.91 (0.46, 1.79)
South Asian	1.56 (0.86, 2.84)	0.81 (0.42, 1.57)
East Asian	0.49 (0.60, 3.47)	0.69 (0.93, 5.17)
Others	0.42 (0.08, 1.98)	0.30 (0.05, 1.62)
Active disease (A or B score in BILAG domains)		
General	8.07 (5.13, 12.71)	2.16 (1.21, 3.87)
Musculoskeletal	2.79 (2.02, 3.84)	1.75 (1.20, 2.55)
Mucocutaneous	2.66 (1.94, 3.66)	2.19 (1.53, 3.14)
Neurological	3.27 (1.79, 5.96)	
Cardiovascular/respiratory	9.15 (4.90, 17.08)	2.26 (1.04, 4.87)
Renal	4.06 (2.72, 6.04)	
Haematological	2.68 (1.95, 3.69)	1.73 (1.19, 2.51)
Other vascular manifestations:		3.28 (2.34, 4.61)
RP	4.67 (3.40, 6.42)	
Livedo reticularis	3.43 (1.35, 8.69)	
Laboratory		
Anti-dsDNA	2.17 (1.57, 3.00)	1.40 (1.01, 1.97)
Low C3/C4	3.12 (2.29, 4.25)	1.85 (1.31, 4.31)
Anti-Ro/Anti-La ever	2.35 (1.48, 3.83)	
Anti-RNP ever	2.00 (1.18, 3.39)	
Anti-Sm ever	3.65 (2.08, 6.41)	2.37 (1.31, 4.31)
Medications		
Prednisolone	0.79 (0.55, 1.15)	0.92 (0.62, 1.36)
Antimalarial	0.50 (0.36, 0.70)	0.65 (0.46, 0.92)
MMF	0.60 (0.36, 1.01)	0.93 (0.51, 1.71)
CYC	2.39 (1.41, 4.04)	1.82 (0.97, 3.40)
AZA	0.53 (0.37, 0.76)	0.62 (0.42, 0.91)

CV: cutaneous vasculitis; HR: hazard ratio.

After 10 years of follow-up, SLE patients with CV were more likely to have one or more items of organ damage compared with those without CV (55.6% *vs* 40.2%, *P* = 0.006); presence of organ damage was higher in both patients with minor CV (61.2% *vs* 45.2%, *P* = 0.006) or major CV (81.8% *vs* 47.2%, *P* = 0.002). In addition, patients with CV had significantly higher frequency of damage in the musculoskeletal (29.2% *vs* 11.8%, *P* < 0.001), skin (9.4% *vs* 2.0%, *P* < 0.001) and peripheral vascular (6.6% *vs* 2.0%, *P* = 0.027) SDI domains. However, the number of patients who had died at 10 years was similar in SLE patients with CV and those without (11.3% *vs* 12.2%, *P* = 0.804) (Table 4).

**Table 4.** Comparison of SDI domains and mortality in patients with and without CV

SDI organs	With CV (*n* = 106) n (%)	Without CV (*n* = 286) n (%)	*P*-value
Ocular	14 (13.2)	27 (9.4)	0.279
Neuropsychiatric	18 (16.9)	33 (11.6)	0.155
Renal	6 (5.6)	8 (2.7)	0.175
Pulmonary	9 (8.4)	13 (4.5)	0.132
Cardiovascular	9 (8.4)	13 (4.5)	0.132
Peripheral vascular	7 (6.6)	6 (2.0)	0.027
Gastrointestinal	3 (2.8)	9 (3.1)	0.872
Musculoskeletal	31 (29.2)	34 (11.8)	<0.001
Skin	10 (9.4)	6 (2.0)	0.001
Diabetes	1 (0.9)	5 (1.7)	0.564
Gonadal failure	4 (3.7)	4 (1.3)	0.143
Malignancy	3 (2.8)	8 (2.7)	0.982
Total damage	59 (55.6)	115 (40.2)	0.006
Mortality	12 (11.3)	35 (12.2)	0.804

CV: cutaneous vasculitis; SDI: SLEDAI damage index.

## Discussion

We have presented the changes in epidemiology of, and risk factors for, CV in a large longitudinal inception cohort of SLE patients followed for up to 20 years in a single centre in the UK. We found that the incidence rates of CV decreased over the duration of follow-up and were associated with defined clinical and serological features.

The overall prevalence of CV in our cohort was 27%. Similarly, in a large multi-ethnic US cohort of over 2500 patients, the prevalence of CV was 17.3% [10] and in a longitudinal Mexican study of 540 patients, 31.5% had CV [7]. Also, a small cross-sectional study reported that 30% of lupus patients had CV [11]. In contrast to the above, a larger cross-sectional Spanish study of 670 patients found a lower prevalence of 10.1% [8]. In this study the majority of patients were of White ethnicity which may suggest a potential ethnic variation, although in our study African or Caribbean ethnicity was only associated with CV in univariate analysis.

This study is the first to outline the incidence rates and cumulative incidence of CV across a 20-year follow-up period. By adopting this approach, we demonstrated a decrease in the incidence rates of CV over the period of follow-up. This is contrary to the findings of Drenkard *et al.* who reported that CV was associated with longer disease duration [7]. This discrepancy could potentially be explained by the differences in the study periods; our analysis extending until 2013 (compared with 1990), capturing the effects of newer SLE treatments. There may also be differences in access to healthcare between patients in Mexico and the UK and variations in defining vasculitis cases (clinical definition with or without biopsy *vs* BILAG definition) and differences in patients’ ethnic backgrounds as we had no patients from Mexico in our study.

There are conflicting data about whether patients from an African or Caribbean background are at increased risk of CV. Kallas *et al.* reported that African American patients had a 20% higher risk of having CV compared with White patients [10] but in our study, being from an African or Caribbean background was only associated with CV in a univariate model. These discrepancies may reflect differences in access to healthcare as all UK residents, including our study population, benefit from equal access to the National Health Service (NHS). Furthermore, we cannot exclude the effects of confounding and multi-collinearity as being from an African or Caribbean background is associated with other variables which remained in our model including anti-dsDNA antibodies, hypocomplementemia and haematological manifestations. No other studies have investigated CV in multiethnic SLE cohorts. In addition, our study included very few patients from East Asia and no Hispanic patients. Consequently, further studies in these populations are warranted.

We found that over 40% of our patients experienced recurrent CV episodes, with 10% having six or more events. Similarly, two studies documented that ∼35% of patients with CV had recurrent episodes at some point during their disease course [7, 10]. This highlights the need to analyse the risk factors in models that can take account of multiple episodes per subject, rather than time-to-first-event analyses. This is the first study to use the multivariable Cox frailty model to identify factors associated with specific disease features in SLE. Unlike traditional survival analyses, which only assess the first incidence of a CV episode per patient, the frailty model employed in this study accounts for the recurrent and time-varying nature of CV episodes and the effects of inter-individual variability on risk [21].

Overall patients with CV were likely to have active disease in other systems at the same time as the CV episode. RP, general (constitutional), mucocutaneous, musculoskeletal, cardiovascular and respiratory, and haematological systems were associated with CV. Several studies have documented that CV was associated with constitutional, mucocutaneous, musculoskeletal and haematological manifestations in SLE patients. Constitutional manifestations [11, 22], discoid rash [10, 23, 24], photosensitivity [24], alopecia, oral ulcers [22], acute cutaneous lupus [22, 24] and myositis [10] have been found to be associated with the development of CV. Moreover, Gheita *et al.* [11] documented an association between CV and constitutional, mucocutaneous and musculoskeletal features. Additionally, some studies reported that haematological manifestations including anaemia [8, 10, 11], positive Coombs test [10], leukopenia [7, 10, 22] and lymphopenia [7] were associated with the development of CV.

Regarding major organ involvement, there are discrepancies in the association between CV and renal, neurological and cardiovascular involvement, with some studies confirming these associations and others not. Using a clinical definition of CV, an Egyptian retrospective study of 50 lupus patients concluded that lupus nephritis and cardiovascular manifestations were significantly linked to the development of CV [11]. Similarly, in a cohort comprising 667 lupus patients, vasculitis (of whom over 87% had CV) was associated with myocarditis, serositis and psychosis [7]. However, Kallas *et al.* [10] used the SLEDAI definition of CV and found no association with cardiorespiratory, renal or neurological involvement. Similarly, Gomes *et al.* [22] reported that renal and neurological involvement was similar in SLE patients with or without digital vasculitis.

A positive association between anti-dsDNA, anti-Sm and hypocomplementemia and CV was reported in our multivariable model, consistent with other studies [7, 8, 10, 11]. However, a few reports did not find an association between hypocomplementemia or anti-dsDNA and CV. There were discrepancies regarding the associations of anti-Ro/SSA and anti-La/SSB with CV, with some studies confirming these associations [8, 11, 25] while others did not observe them [10, 26].

This study is the first to look at the effect of medications on the development of CV. There was a negative association between CV and concurrent use of AZA, and antimalarials, suggesting a protective effect. However, a positive association between CYC and CV was documented in our univariate model, but not in the multivariable model, in which disease activity was a cofounder. This association could be explained by the possibility/use of CYC to treat patients with severe and refractory CV patients (confounding by indication). CYC has been widely used in treatment of severe, life threatening complications of lupus including LN and systemic vasculitis [27], particularly before the availability of MMF.

Lupus patients with minor or major CV were more likely to have at least one item of organ damage compared with those without CV and patients with CV were more likely to have damage in the musculoskeletal, skin and cardiovascular systems. Previous reports documented that SLE patients with CV tended to experience higher damage and disease activity [10, 11]. CV may therefore be a useful marker of patients who are likely to have higher overall disease activity and be at higher risk of organ damage. However, we did not find an association between death and CV. Similarly, Drenkard *et al.* [7] reported that CV was not associated with increased mortality in lupus patients.

A key strength of our study is that we include only inception patients which enable a better understanding of the early course of SLE, decrease the risk of any potential bias, and reduce the impact of any previously unrecorded event on the outcome of the study. This is highlighted by our observation that CV tends to occur early in the disease course. This is also the first study to account for multiple episodes of CV per patient in a statistical model.

A limitation of our study is that we were unable to include LA and aCL antibodies in our analysis as the assays changed several times over the follow-up period. Furthermore, our patients were recruited within 3 years of achieving the fourth ACR criterion for lupus. While a shorter time frame would be more precise, this would reduce the number of eligible patients for the study [15]. In addition, as there was a decrease in the number of patients followed beyond 10 years, and there were fewer CV events observed later in the study, the CIs are wide and the incidence rates estimates may be less precise in the later follow-up period. As the study endpoint was set at 2013, the analysis of the risk factors did not include the impact of newer biological treatments such as rituximab and belimumab.

In summary, our study represents one of the largest multi-ethnic inception cohorts of patients with SLE population in the UK and has identified key factors associated with the development of CV and a decrease in the cumulative incidence of CV over the study period. We propose that CV is a severe manifestation of active disease and is associated with increased likelihood of organ damage. Intriguingly, AZA and antimalarials may have protective effect against the development of CV.

## Supplementary Material

keae672_Supplementary_Data

## Data Availability

These data form part of an ongoing study. Upon study completion, these data in anonymized format may be available upon reasonable request to the corresponding author.
