# Supplementary material for: Cutaneous vasculitis in systemic lupus erythematosus: epidemiology and risk factors over a 20-year follow-up
Source: Rheumatology (Oxford). 2024 Dec 11;64(5):2749–55. doi: 10.1093/rheumatology/keae672 (PMC12048080; doi:10.1093/rheumatology/keae672)
Supplement: keae672_Supplementary_Data [file keae672_supplementary_data.docx]

**Supplementary materials**

**Supplementary table 1: Estimated cumulative incidence of CV in triannual intervals over the period of follow-up**

| Period of follow-up in years | CV Cumulative incidence (95% CI) |
| --- | --- |
| **3** | 0.17 (0.13, 0.21) |
| **6** | 0.23 (0.19, 0.27) |
| **9** | 0.27 (0.23, 0.32) |
| **12** | 0.29 (0.24, 0.34) |
| **15** | 0.29 (0.25, 0.35) |
| **18** | 0.33 (0.27, 0.40) |
| **>18** | 0 |

The cumulative incidence for the first episode of CV was calculated using 1-KM survival function at each 3-year time point

**Supplementary table 2: Shoenfeld residuals test for Proportional Hazard assumptions of the multivariable shared frailty Cox model**

| Variables | chi square | P value |
| --- | --- | --- |
| **Sex**: male | 1.16 | 0.28 |
| **Age** | 0.50 | 0.47 |
| **Ethnicity**: |  |  |
| African or Caribbean | 4.61 | 0.03 |
| South Asian | 0.35 | 0.55 |
| East Asian | 0 | 0.97 |
| Others | 0.06 | 0.80 |
| **BILAG domains** |  |  |
| General | 0.57 | 0.44 |
| Musculoskeletal | 1.38 | 0.24 |
| Mucocutaneous | 0.26 | 0.60 |
| Cardiovascular/respiratory | 0.58 | 0.44 |
| Haematological | 3.86 | 0.04 |
| Other vascular manifestations:   - Raynaud’s phenomenon | 0.07 | 0.79 |
| **Laboratory** |  |  |
| Anti-dsDNA | 0 | 0.97 |
| Low C3 / C4 | 0.01 | 0.93 |
| Anti-Sm ever | 0.45 | 0.50 |
| **Medications** |  |  |
| Prednisolone | 0.13 | 0.71 |
| Antimalarial | 4.32 | 0.04 |
| Mycophenolate mofetil | 0.42 | 0.51 |
| Cyclophosphamide | 0 | 0.97 |
| Azathioprine | 0.42 | 0.51 |
| Global | 22.9 | 0.28 |

AIC= 3554.7 BIC= 3700.9


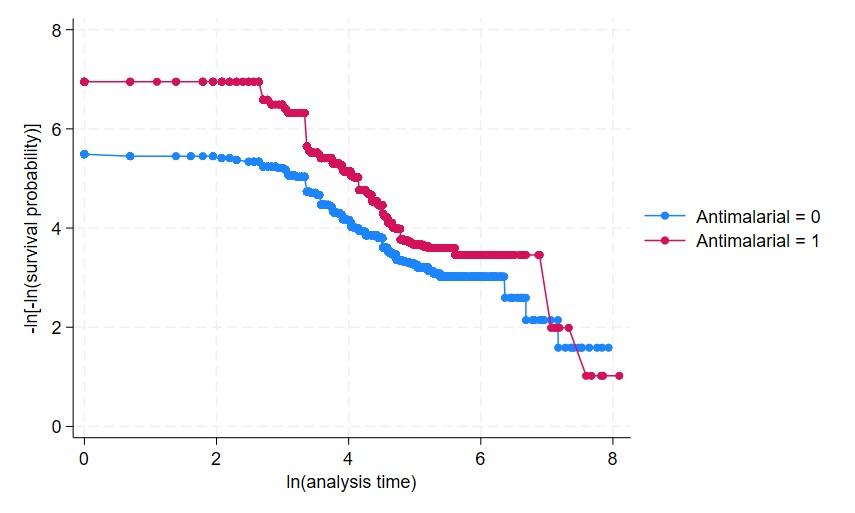


**Figure (1) Schonfeld residuals test plot for antimalarial treatment**. Red line: Patients receiving antimalarial medication (Antimalarial =1). Blue line: Patients not receiving antimalarial medication (Antimalarial =0). The graph shows a potential violation of the Proportional Hazards assumption, suggesting that the effect of antimalarial medication on the hazard estimates is time dependent.


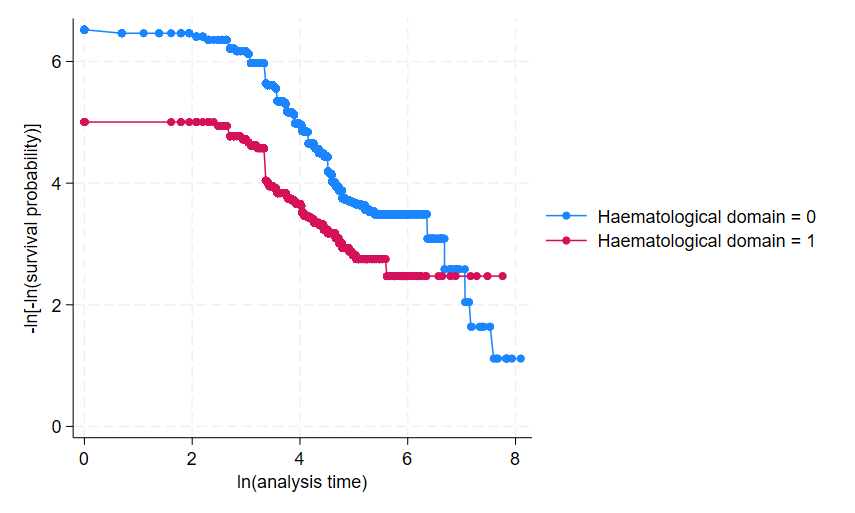


**Figure (2) Schonfeld residuals test plot for Haematological domain of classic BILAG**. Red line: Patients with active SLE in the Haematological domain of classic BILAG (Haematological domain= 1). Blue line: Patients without active SLE in the Haematological domain of classic BILAG (Haematological domain= 0). The graph shows a potential violation of the Proportional Hazards assumption, suggesting that the effect of antimalarial medication on the hazard estimates is time dependent.

**Supplementary table 3:** multivariable Cox Proportional Hazard frailty model with TVC.

| Variables | Multivariable cox regression HR (95% CI) |
| --- | --- |
| **Sex**: male | 0.57 (0.16, 2.00) |
| **Age** | 0.95 (0.93,0.97) |
| **Ethnicity**: White |  |
| African or Caribbean | 0.91 (0.46, 1.80) |
| South Asian | 0.85 (0.44,1.66) |
| East Asian | 0.69 (0.93, 5.15) |
| Others | 0.31 (0.05, 1.66) |
| **Active disease (A or B score in BILAG domains)** |  |
| General | 2.12 (1.18, 3.80) |
| Musculoskeletal | 1.74 (1.19, 2.52) |
| Mucocutaneous | 2.20 (1.54, 3.15) |
| Neurological |  |
| Cardiovascular/respiratory | 2.16 (0.99,4.70) |
| Renal |  |
| Haematological | 2.28 (1.34, 3.85) |
| Other vascular manifestations:   - Raynaud’s phenomenon - Livedo reticularis | 3.27 (2.33, 4.60) |
| **Laboratory** |  |
| Anti-dsDNA | 1.40 (1.00, 1.96) |
| Low C3 / C4 | 1.84 (1.30, 2.61) |
| Anti-Ro /Anti-La ever |  |
| Anti-RNP ever |  |
| Anti-Sm ever | 2.38 (1.31, 4.33) |
| **Medications** |  |
| Prednisolone | 0.91 (0.62, 1.35) |
| Antimalarial | 0.55 (0.37, 0.82) |
| Mycophenolate mofetil | 0.95 (0.51, 1.73) |
| Cyclophosphamide | 1.77 (0.94, 3.33) |
| Methotrexate |  |
| Azathioprine | 0.62 (0.42, 0.91) |
| **TVC** |  |
| Haematological | -0.004 (-0.010, 0.002) |
| Antimalarial | 0.001 (-0.001, 0.004) |

AIC= 3550.3 BIC= 3711.1
